# Quantitative Mass Spectrometry Imaging Protocols for Spatially Heterogeneous Samples

**DOI:** 10.1021/acs.analchem.5c00677

**Published:** 2025-05-22

**Authors:** Reza Shariatgorji, Michael Niehues, Anna Nilsson, Tina Angerer, Nikolas Stroth, Wojciech Paslawski, Sandra Jabre, Per Svenningsson, Per E. Andrén

**Affiliations:** † Department of Pharmaceutical Biosciences, Spatial Mass Spectrometry, Science for Life Laboratory, Uppsala University, Uppsala SE-75124, Sweden; ‡ Department of Clinical Neuroscience, 27106 Karolinska Institute, Stockholm SE-17177, Sweden

## Abstract

Matrix-assisted laser desorption/ionization mass spectrometry imaging (MALDI-MSI) is a promising tool for the spatial quantitation of endogenous and exogenous compounds directly in biological tissue sections. However, precise quantitation may be hampered due to matrix effects and variations in ionization efficiency, especially in spatially heterogeneous samples such as brain tissue. In this study, we developed and implemented two advanced MALDI-MSI protocols to address these limitations by employing a standard addition approach. The protocols involved the homogeneous spraying of standard solutions onto tissue sections to minimize the matrix effects associated with heterogeneous samples. The first method utilized spraying of deuterated analogues of neurotransmitters across all tissue sections for normalization, while calibration standards were applied in a quantitative manner to consecutive tissue sections. The second method employed two stable isotope-labeled compounds: one for calibration and the other for normalization. Both methods were applied to quantify neurotransmitters and their metabolites, e.g., dopamine, norepinephrine, and 3-methoxytyramine, in rodent brain tissue. The results showed strong linearity between signal intensities and analyte concentrations across brain tissue sections with values comparable to those obtained using high-performance liquid chromatography-electrochemical detection. The standard addition approach significantly enhanced the quantitation accuracy by accounting for tissue-specific matrix effects, providing a robust method for the spatial quantification of neurotransmitters in complex brain tissue environments.

## Graphical abstract

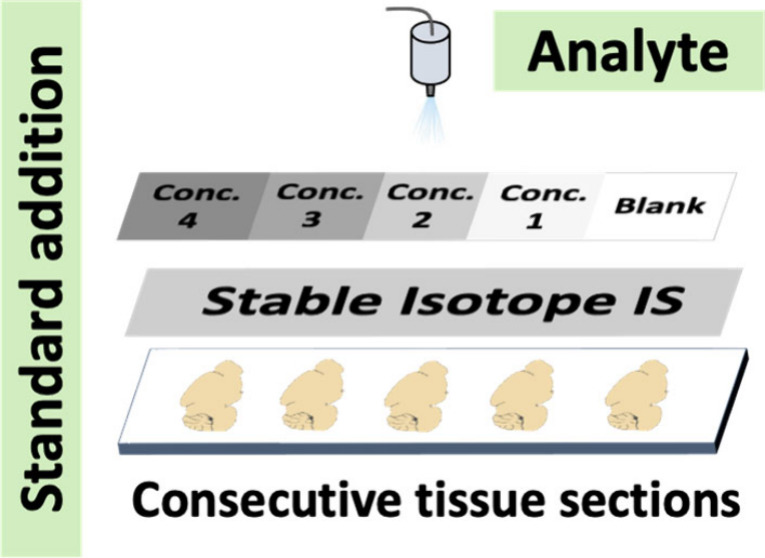

Mass spectrometry imaging (MSI) methods, namely matrix-assisted laser desorption/ionization (MALDI) and desorption electrospray ionization (DESI), are powerful label-free imaging tools capable of generating comprehensive molecular maps directly from biological tissue sections, exhibiting high sensitivity and near to cellular lateral resolution.^1^ MSI has been shown to be a potentially useful technique for the spatial quantitative analysis of small molecules across tissue sections.^2–5^ However, achieving precise quantitation in MSI necessitates comprehensive control over matrix effects, which may have a detrimental effect on the ionization process, as well as extraction yield of the analyte of interest from the tissue. MSI protocols are mainly optimized for fresh frozen, untreated tissue sections, which naturally contain cell debris, salts, and other ionization-suppressing molecules that can affect the precision, accuracy, and reproducibility of quantitation. The matrix effects tend to reduce the analytical signal, which may result in significant errors. Several strategies, including tissue extinction coefficient normalization,^6^ spiked homogenates as mimetic tissue models,^7,8^ wide isolation MS/MS imaging^9^ and normalization against a stable isotope-labeled (SIL) compound,^10^ have been developed to address the above-mentioned problems.

Current quantitative MSI protocols commonly rely on spotting known concentrations of an analyte on control tissue sections and extracting the signals to generate a calibration curve.^3,4^ This strategy is challenging since tissue samples analyzed by MSI are often inhomogeneous with matrix effects varying spatially across the tissue sections. For example, brain tissue is composed of both gray and white matter, each containing distinct micro- and macrostructures with varying concentrations and types of endogenous compounds. Chemical variations between regions, such as densely packed neurons in gray matter and myelinated axons in white matter, contribute to the significant heterogeneity across the brain. This inherent chemical complexity can lead to uneven lateral matrix effects during analysis and is exacerbated by local suppression effects.^11,12^ These issues present a particular challenge when calibration standards, which are often manually pipetted with typical spot sizes of around 1 mm in diameter, are applied to brain regions different from those targeted for quantitation. In such cases, any mismatch between the composition at the calibration spot and specific brain structures of interest may introduce inaccuracies in the quantification of the analytes.

The standard addition method is an analytical chemistry technique typically used for the quantitation of complex liquid samples containing multiple interfering components that may cause matrix effects.^13^ The method involves measuring the signal of an analyte of interest in a sample compared with that of the analyte spiked into the sample with varying amounts of known standards added. The concentration of the analyte is then calculated from a least-squares analysis of the intensity vs concentration of added analyte and is represented by the *x*-intercept of the line.

The method has been frequently used to eliminate/reduce matrix effects for different analytical techniques, mostly those dealing with liquid phase (homogeneous) analytical samples.^14^ The standard addition method has previously been applied to MSI using multiple isotopically labeled internal standards to achieve per-pixel calibration. However, our current approach employs consecutive tissue sections, significantly reducing the requirement to a single labeled analogue solely for normalization purposes.^15^ To address the challenges with quantitation listed above, we adapted the standard addition method for use with MSI by spraying standards onto mouse brain tissue sections (Supporting Information) instead of spiking them into solution. Because the protocol involved reproducible quantitative homogeneous application of the analyte to the sample surface used for MSI, it had the advantage of being spot-free.

Two different quantification approaches, including standard addition based on spraying rather than spotting calibration standards onto brain tissue sections, were employed for the measurement of endogenous neurotransmitters (Figure 1). A robotic sprayer (TM-sprayer, HTX-Technologies LLC, Chapel Hill, NC, USA) with known quantitative hardware parameters was used to apply the standard solutions. The density of the deposited standard was calculated from the following equation: $w=\frac{n\times C\times F}{V\times d}$ , where *w* is the compound density, *n* is the number of passes applied, *C* is the concentration of solution sprayed over the tissue section, *F* is the flow rate of the spraying solution, *V* is the velocity of the sprayer head and *d* is the spacing between each track.

**Figure 1:**
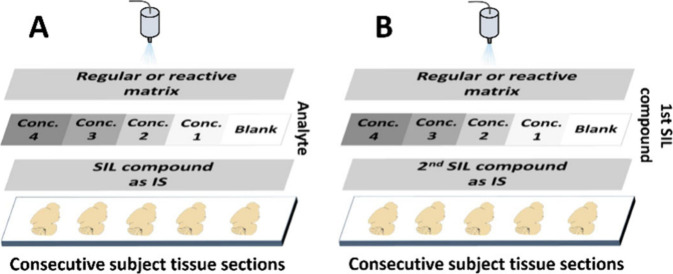
Standard addition sample preparation. Several consecutive brain tissue sections were placed on ITO slides for both methods. (A) In method A, standard isotope labeled (SIL) reference compounds (as internal standards) were sprayed over all tissues for normalization purposes. Afterward, standard solutions containing the target analyte at different concentrations were applied over each individual tissue section, followed by spraying of the reactive matrix. (B) Method B (internal calibration method using SIL compounds) was similar to method A, except that a second SIL compound at different concentrations was used as the calibration standard instead of addition of the target analyte(s).

Sagittal brain tissue sections (12 μm thickness) were placed on ITO coated slides as centrally as possible with a sufficient distance between each section to avoid cross-contamination. Stock solutions of neurotransmitters and corresponding SIL analogues, including dopamine (DA) (1 mM), DA-*d*_4_ (5 mM), DA-(^13^ *C*)_6_ (1 mM), 3-methoxytyramine (3-MT) (0.1 mM), 3-MT-*d*_3_ (0.1 mM), norepinephrine (NE) (0.1 mM), NE-*d*_6_ (0.1 mM), 5-hydroxytryptamine (5-HT) (0.1 mM), and 5-hydroxyindoleacetic acid (5-HIAA) (0.1 mM) were dissolved in 0.1 N HCl/50% MeOH (1/9 v/v) and sonicated for 20 min. Solutions including calibration standards and SIL compounds for normalization were diluted in 50% MeOH and applied with the robotic sprayer using the following parameters: nozzle temperature, 90 °C; solvent flow rate, 70 μL/min; nozzle velocity, 1100 mm/min; nitrogen gas pressure, 6 psi; track spacing, 2.0 mm. To validate the analytical precision and accuracy of the sprayer as a calibration tool for depositing calibration standards, a known concentration of 9-aminoacridine (5 mg/mL) was sprayed onto the slide in six replicates using the above-mentioned conditions. The theoretical amount of matrix deposition per surface area (0.095 mg/cm^2^) closely matched the experimentally determined density (0.092 ± 0.006 mg/cm^2^, n = 6), which was measured gravimetrically by weighing the slides before and after spraying.

SIL internal standards (DA-*d*_4_, 3-MT-*d*_3_ and NE-*d*_6_ for method A and DA-*d*_4_ for method B) were sprayed over sagittal mouse brain tissue sections in six passes to achieve a concentration of 7.2 pmol/mg tissue. For both methods, samples were stored in a vacuum desiccator for 10 min to dry before application of the calibration standards. In method A, different concentrations of calibration standards, i.e., DA, 3-MT, NE, 5-HT and 5-HIAA, were sprayed in four passes over the intended brain tissue sections, whereas the other tissues were covered by a coverslip (Figure 2). Samples for method B were prepared in a similar way as method A, except DA-(^13^ *C*)_6_, 3-MT-*d*_3_ and NE-*d*_6_ were used as calibration standards (Figure 3).

**Figure 2:**
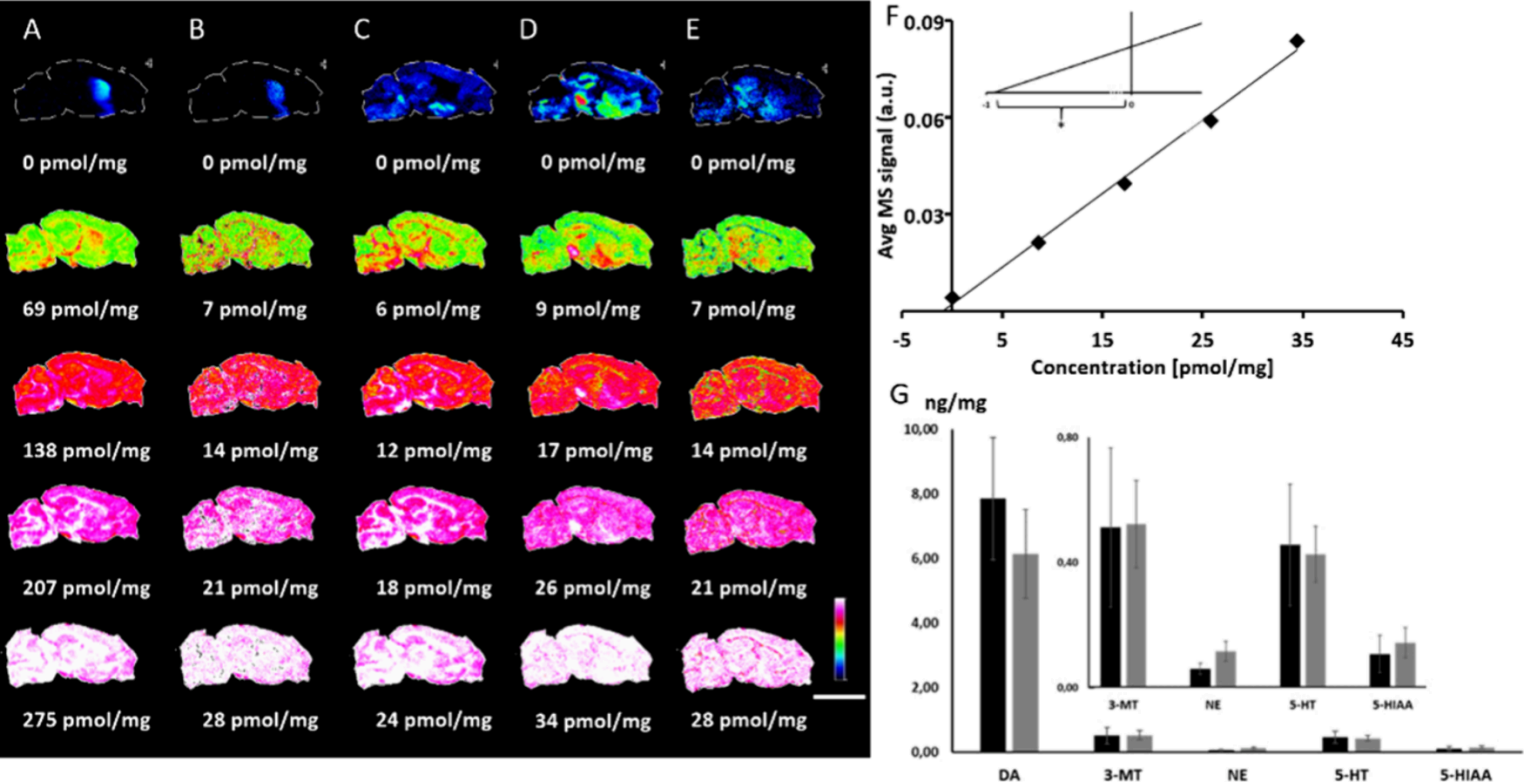
Quantitative mass spectrometry imaging of neurotransmitters using method A. Standard addition was used for quantitation of neurotransmitters in AMPT-treated mouse tissue sections. Standard solutions of DA-*d*_4_, 3-MT-*d*_3_, and NE-*d*_6_ were sprayed over consecutive sagittal mouse brain tissue sections for normalization. Tissue sections were then covered by different concentrations of standards (A) DA, (B) 3-MT, (C) NE, (D) 5-HT, and (E) 5-HIAA. (F) Extracted average MS signals showed a high level of linearity (*R* ^2^ = 0.995), as exemplified for 5-HT. (G) Absolute values of quantities for DA, 3-MT, NE, 5-HT, and 5-HIAA in the striatal structure of the brain acquired by this method (black bars) were in good agreement with average quantities determined by HPLC-ECD (gray bars). The Y-axis represents concentration (ng/mg brain tissue). Data are shown by using a rainbow scale (representing the ion intensity) for visualization. Scale bar 5 mm; lateral resolution 100 μm. Abbreviations: AMPT, α-methyl-p-tyrosine; DA, dopamine; 3-MT, 3-methoxytyramine; NE, norepinephrine; 5-HT, 5-hydroxytryptamine; 5-HIAA, 5-hydroxyindoleacetic acid.

**Figure 3:**
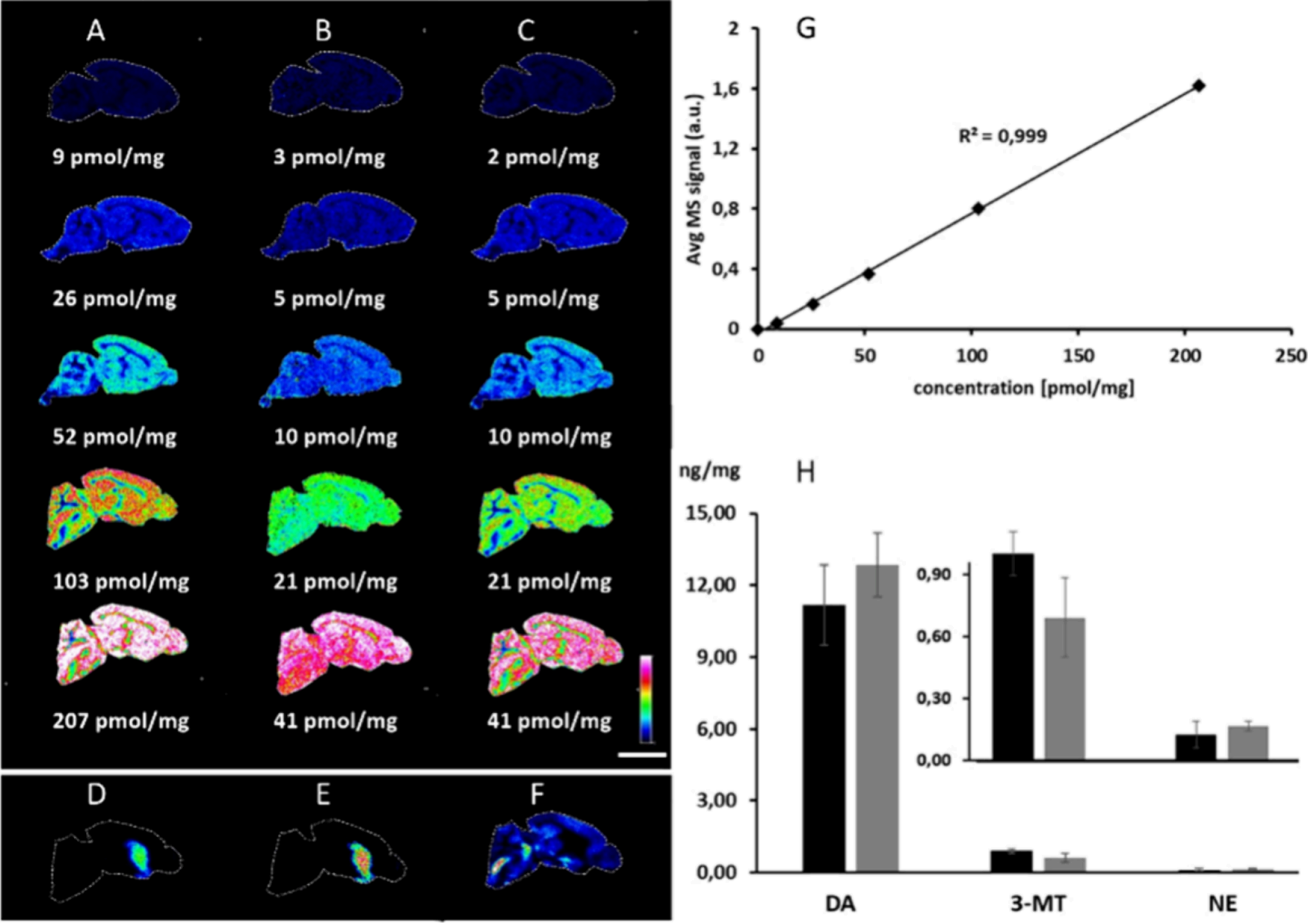
Quantitative mass spectrometry imaging of neurotransmitters using method B. Sagittal mouse brain tissue sections were covered with DA-*d*_4_ for normalization. Afterward, stable isotope labeled standards (A) DA, (B) 3-MT, and (C) NE were sprayed over the tissue sections with different concentrations to quantitate (D) endogenous DA, (E) 3-MT, and (F) NE. (G) Extracted average MS signals showed a significantly high level of linearity (*R* ^2^ = 0.999), as exemplified for DA. (H) Absolute values of quantities for DA, 3-MT, and NE in the striatal structure of the brain acquired by this quantitation method (black bars) were in agreement with the average quantities determined by HPLC-ECD (gray bars). The Y-axis represents concentration (ng/mg brain tissue). Data are shown using a rainbow scale (representing the ion intensity) for visualization. Scale bar 5 mm, lateral resolution 100 μm. Abbreviations: DA, dopamine; 3-MT, 3-methoxytyramine; NE, norepinephrine.

Optical images of the samples were recorded using a flatbed scanner (Epson perfection V500, Epson). The derivatizing MALDI matrix FMP-10 was prepared as previously described.^16^ In brief, 20 passes of 4.4 mM FMP-10 in 70% acetonitrile were sprayed in horizontal lines over the samples with a solvent flow rate of 80 μL/min, nozzle temperature of 90 °C, nozzle velocity of 1100 mm/min, nitrogen gas pressure of 6 psi, and track spacing of 2 mm.

All MALDI-MSI experiments were performed using a MALDI-FTICR-MS instrument (Solarix XR 7T-2Ω, Bruker Daltonics, Bremen, Germany) equipped with a Smartbeam II 2 kHz Nd:YAG laser. Data acquisition was conducted by using ftmsControl and flexImaging (Bruker Daltonics). The laser power was optimized at the start of each analysis. Samples were analyzed in the positive ion mode using the quadrature phase detection (QPD) (2ω) mode over a mass range of *m*/*z* 150–1500. Spectra were recorded by summing signals from 100 laser shots per pixel. A matrix derived peak at *m*/*z* 555.2231 was used as a lock mass for the internal *m*/*z* calibration. Red phosphorus was used for external calibration of the method. Acquired data were converted to imzML format by flexImaging and then to msIQuant^17^ format for quantitative analysis.

Two different methods, both based on spraying internal standards for both normalization and calibration, were used for quantitation of neurotransmitters in mouse brain tissue sections from animals treated with saline or α-methyl-*p*-tyrosine (AMPT). AMPT is a tyrosine hydroxylase inhibitor that blocks the enzyme tyrosine hydroxylase, the rate-limiting enzyme in the synthesis of catecholamines, such as dopamine, norepinephrine, and epinephrine. When tyrosine hydroxylase is inhibited by AMPT, the production of these neurotransmitters is reduced.^18^ The first method was based on application of target analytes to consecutive tissue sections in a quantitative manner, while labeled analogues of the analytes were sprayed over the whole slide for normalization. Catecholamines, including NE, DA and its metabolite 3-MT, and the indolamine neurotransmitter 5-HT and its metabolite 5-HIAA (Figure 2a-e), were sprayed over tissue sections at different concentrations, whereas a blank tissue section that was covered throughout the spraying cycles was employed to represent the endogenous concentration of the corresponding neurotransmitter. Signal intensity values of the neurotransmitters and metabolites were extracted from the striatal structure of the brain and the data were plotted against the amounts of added analytes. The calculations exhibited strong linearity, achieving values of *R* ^2^ greater than 0.99 (Figure 2f). The endogenous concentration of each analyte was obtained from the intersection of the trend line with the *x*-axis (Figure 2f). A well-established quantitative method using HPLC-ECD (Supporting Information) was employed to quantify neurotransmitters in the selected brain structure of the mouse brain (striatum) (Table S1). The MALDI-MSI results obtained using the standard addition protocol aligned closely with those acquired by HPLC-ECD (Figure 2g). However, note that there was a concentration gradient of neurotransmitters across the brain tissue sections. Hence, the anatomical level or thickness of the sample collected for HPLC analysis may not have corresponded exactly to that of the imaging analysis region. These factors introduced variability into the measurements, making it unsuitable to perform a direct statistical comparison between the two methods.

The standard addition method offers significant advantages over external calibration methods by negating the requirement for a separate reference standard and addressing complications arising from matrix effects, chemical interference, and instrument response drift. The traditional external calibration method, which typically involves manually spotting calibration standards onto tissue sections, is limited by the difficulty of applying small but precise and reproducible spots (∼0.2 μL) over the different microstructures of brain tissue, such as white and gray matter. This spatial inhomogeneity makes it difficult to achieve reproducibility, potentially introducing substantial errors in quantification. While robotic spotters may offer more precise control over standard deposition, their high cost, complexity, and handling requirements make them less practical for widespread use.

In contrast, standard addition protocols utilizing a homogeneous spraying technique allow for a more even distribution of standards across the tissue surface, reducing the variability caused by spatial heterogeneity. This approach can enhance the accuracy and reproducibility of quantifying neurotransmitters and other analytes within heterogeneous tissue samples, making it a more robust solution for addressing matrix effects.

In addition, we developed a second quantitation method based on spraying the standards, but in this approach, two different SIL compounds were used. One set was dedicated to calibration standards, whereas the other was used for normalization (Figure 3). This protocol was applied for the quantitation of DA, 3-MT, and NE (Figure 3a–f) in the striatal structure of brain tissue sections of saline-treated mice. The resulting images showed that the ion intensities of the same concentration of standards sprayed over the tissue sections varied across different brain structures (Figure 3a–c). This variation was due to the different chemical compositions of the brain regions, which affected the ionization and desorption yields, leading to differences in the ion intensities for the same concentration. Application of a standard addition protocol that used a calibration curve from a specific brain structure to quantify the analyte within the same structure helped address these challenges. The data showed a high degree of linearity (*R* ^2^ > 0.99) between signal intensity values extracted from the striatal structure of the brain and the amount of sprayed SIL compound (Figure 3g). Unlike that in the first method, the trend line intersected at the origin of the graph (Figure 3g). The extraction efficiency may differ between endogenous analytes within tissue and externally applied standards. To minimize this, we utilized consecutive tissue sections for standard addition, isotopically labeled standards for normalization, and multiple heated spray passes of standards and reactive matrices to improve tissue penetration.

The concentrations of DA, 3-MT, and NE quantified by the latter method agreed with values obtained from HPLC-ECD analysis (Figure 3h) (Table S1), although the neurotransmitter concentrations varied across the tissue section. Hence, the anatomical level and thickness of the sample collected for HPLC-ECD may not have exactly matched those of the imaging region.

## Supplementary Material



## Notes

Detailed descriptions of the experimental procedures are provided, including the chemicals and reagents utilized in this study, animal experiments conducted, and HPLC-ECD method employed for the determination of neurotransmitters (PDF)
