# Supplementary material for: Quantitative Mass Spectrometry Imaging Protocols for Spatially Heterogeneous Samples
Source: Anal Chem. 2025 May 22;97(21):10957–61. doi: 10.1021/acs.analchem.5c00677 (PMC12138873; doi:10.1021/acs.analchem.5c00677)
Supplement: Supplementary file 1 [file ac5c00677_si_001.pdf]

SUPPORTING INFORMATION

Quantitative mass spectrometry imaging protocols for spatially heterogeneous samples

Reza Shariatgorji†, Michael Niehues†, Anna Nilsson†, Tina Angerer†, Nikolas Stroth‡, Wojciech Paslawski‡, Sandra Jabre‡, Per Svenningsson‡, Per E. Andren†*

† Department of Pharmaceutical Biosciences, Spatial Mass Spectrometry, Science for Life Laboratory, Uppsala University, Uppsala SE-75124, Sweden

‡ Department of Clinical Neuroscience, Karolinska Institute, Stockholm SE-17177, Sweden

* Corresponding author: per.andren@uu.se

	SUPPORTING INFORMATION CONTENT	Page
1.	Methods	Page S2
1.1	Chemicals and reagents	Page S2
1.2	Animal experiments	Page S2
1.3	HPLC-ECD determination of neurotransmitters	Page S2
1.4	Table S1. Quantitative results corresponding to Figures 2 and 3	Page S3

1. Methods

1.1 Chemicals and reagents

All chemicals were purchased from Sigma-Aldrich (Stockholm, Sweden) and used without further purification unless otherwise stated. The reactive matrix 4-(anthracen-9-yl)-2-fluoro-1-methylpyridin-1-ium iodide (FMP-10) was purchased from Tag-ON AB (Uppsala, Sweden). All labeled standards were purchased from CDN Isotopes (Quebec, Canada).

1.2 Animal experiments

C57BL/6 male mice were kept in rooms under controlled conditions of 12-h light/dark cycles, temperature and humidity, with food and water provided ad libitum. The experiments were performed in agreement with the European Council Directive (86/609/EEC) and approved by the local Animal Ethics Committee (N40/13, Stockholms Norra Djurförsöksetiska Nämnd). Efforts were made to minimize suffering and the number of animals used.

There were two treatment groups (n=4 per group). Mice were sacrificed 4 h after administering saline or α-methyl-*p*-tyrosine (AMPT) (250 mg/kg, i.p.).

Mouse brains were dissected out and separated into two hemispheres by one sagittal cut. One hemisphere was frozen in dry-ice cooled isopentane and stored at -80 °C until cryo-sectioning prior to MSI analysis. The other hemisphere was used for immediate dissection of brain regions. Dissected brain regions were quickly weighed, frozen immediately and stored at -80 °C until analysis by high-performance liquid chromatography (HPLC) with electrochemical detection (ECD).

1.3 HPLC-ECD determination of neurotransmitters

To perform HPLC-ECD analysis, ice-cold 0.1 M perchloric acid (PCA) was added to tissue samples (50 µL PCA per 10 mg of tissue). Samples were incubated on ice for 10 min, then vortexed and centrifuged at 16000 x g for 10 min at 4 °C. Resulting supernatants were filtered through 0.2 µm nylon membrane inserts and centrifuged at 4000 x g for 5 min. Eluents were immediately stored at -80 °C and subjected to HPLC-ECD analysis within 1 week.

To construct calibration curves, standard solutions of DA, 3-MT, NE, 5-HT and 5-HIAA were prepared in 0.1 M PCA to obtain final standard concentrations of 200, 100, 50, 10, 5, 2 and 1 ng/ml. A Dionex Ultimate 3000 series (Dionex, ThermoFisher Scientific, USA) HPLC-ECD system equipped with a Dionex C18 reversed-phase MD-150 column (3.2 mm x 250 mm, 3 µm particle size) was used for analysis. The column and analytical cell were kept at 30 °C.

The mobile phase consisted of 75 mM monobasic sodium phosphate, 2.2 mM 1-octanesulfonic acid sodium salt, 100 µL/l triethylamine, 25 µM ethylene-diamine-tetra-acetic acid disodium salt and 10 % acetonitrile (v/v), with the pH adjusted to 3.0 using 85% phosphoric acid. For detection of neurotransmitters and metabolites, the voltages of the first and second analytical cells were set to -100 mV and +300 mV, respectively. Processed tissue samples were thawed on ice in the dark for about 1 h before analysis, placed in the autosampler and kept at 5 °C before injection (flow rate 0.4 ml/min, injection volume 20 µl). Chromatograms were recorded using Dionex Chromeleon 7 software during an acquisition time of 55 min.

Table S1. MALDI-MSI and HPLC-ECD quantitative results from biological replicates (n=4) corresponding to method A (Fig. 2) and B (Fig. 3).

A

DA	DA	3-MT	3-MT	NE	NE	5-HT	5-HT	5-HIAA	5-HIAA
MSI	HPLC-ECD	MSI	HPLC-ECD	MSI	HPLC-ECD	MSI	HPLC-ECD	MSI	HPLC-ECD
8,810	7,165	0,880	0,698	0,070	0,117	0,760	0,526	0,180	0,193
4,910	4,951	0,260	0,408	0,040	0,099	0,490	0,477	0,140	0,158
7,650	5,877	0,290	0,471	0,040	0,098	0,260	0,328	0,070	0,117
10,060	7,103	0,610	0,591	0,080	0,168	0,310	0,415	0,030	0,093

B

DA	DA	3-MT	3-MT	NE	NE
MSI	HPLC-ECD	MSI	HPLC-ECD	MSI	HPLC-ECD
11,200	14,561	1,050	0,713	0,120	0,165
9,180	12,371	1,050	0,676	0,110	0,078
12,390	11,535	0,650	0,500	0,114	0,140
12,000	12,983	0,850	0,591	0,126	0,226
